# Reduce cancer inequity and inequality to reduce cancer mortality

**DOI:** 10.1016/j.lanepe.2023.100591

**Published:** 2023-02-01

**Authors:** The Lancet Regional Health - Europe

In February, 2023, World Cancer Day (February 4) and International Childhood Cancer Day (February 15) will be commemorated with “Close the Care Gap” and “Better Survival” campaigns, respectively. The emphasis of these campaigns is to reduce cancer mortality and increase survival. To achieve these aims, it is essential to bridge gaps in cancer inequality—uneven distribution of resources and cancer inequity—unjust, avoidable differences in care or outcomes. The difference between inequality and inequity might seem subtle, but it is not: providing everyone with equal resources (ie, equality) will not close the gap in cancer care unless everyone receives the care they need (ie, equity) to achieve similar outcomes.

Considerable progress has been made towards improvement of cancer survival, largely due to the advances in medical research, rollout of screening programmes, and availability of treatments. However, such progress has also highlighted inequities and inequalities in cancer prevention, diagnosis, and treatment. Factors that contribute to cancer inequality often occur as a result of unequal distribution of resources, whereas the root causes of inequity are due to race, refugee status, poverty, socioeconomic status, gender norms and discrimination, and barriers for minority ethnic populations and people with disability. Cancer inequality is a more commonly used term even when referring to cancer inequities, and thus, often there is little awareness and understanding about the distinction between equity and equality. It is imperative to address both cancer inequity and cancer inequality to improve cancer survival.

Globally, Europeans are disproportionately affected by cancer. Although Europeans account for only 10% of the global population, 25% of all annual cancer cases occur in Europe and cancer is the number one cause of death among Europeans aged younger than 65 years. A study by Vaccarella and colleagues found that within and across countries in Europe, cancer mortality rates were substantially affected by inequities in socioeconomic education status. Individuals with lower education had higher mortality rates than those with higher education across nearly all cancer types. Sex differences were also identified, whereby 32% of cancer deaths in men and 16% in women were associated with educational inequalities. Literacy is a modifiable cancer risk factor, and this study highlights the need to address literacy as one of the causes that is compounding cancer inequity and mortality.

In Europe, one in every 300 children born in 2020 is likely to develop cancer by the age of 19 years. Childhood cancer can lead to substantial short-term and long-term inequalities since cancer is a likely re-occurrence in adult survivors who had childhood cancer. A study by Chang and colleagues showed that the most deprived adult survivors of childhood cancer had a higher burden of health diseases than the least deprived individuals. Furthermore, childhood cancer survivors face difficulties in employment late in life. Frederiksen and colleagues found that by the age of 30 years old, 9.2% of childhood cancer survivors were unemployed for health reasons. From a scientific perspective, childhood cancers remain rare and therefore it is important to collect more data across countries. The new childhood incidence data section of the European Cancer Information System brings these data together from across Europe, which will enable policy makers and stakeholders in EU Member States to better monitor trends and outcomes for different diagnostic groups of childhood cancer.

Inequitable access to cancer screening and treatments further accentuates cancer mortality. Although many European countries have cancer screening programmes, disparities exist by economic status. A study by Bozhar and colleagues found that income-based inequality in breast and colorectal screening was highest in southern Europe, and individuals with a low household income were less likely to utilise cancer screening than those with a high household income. Furthermore, many people cannot access life-saving treatments such as chemotherapy, due to the absence of universal health coverage and costs. Cancer survival rates among people living in rural areas are lower than people living in urban areas due to inaccessibility of resources. To close this inequity gap, uniform population-based screening policies need to be targeted and tailored to the most vulnerable groups in society and mobile services should be provided for individuals living in remote areas.

Collectively, inequities in the prevention, diagnosis, and treatment of cancer can be reduced by strengthening primary health care delivered in communities; equipping health-care professionals with skills and knowledge about how inequity influences cancer care; and implementing policies and programmes with more resources allocated to the most disadvantaged groups. Tackling inequities and inequalities is also an initiative of the EU's Cancer Beating Plan, which addresses cancer disparities associated with countries, age, sex, income, education, and urbanisation and led to the setup of the European Cancer Inequalities Registry. The EU's Cancer Beating Plan is ambitious but highlights the issue of cancer inequality and inequity for many governments, which should be commended. As the European population gets older, the number of patients with cancer will increase in the future. Both inequity and inequality represent different facets of cancer care disparities that need to be addressed separately, since a one-size-fits-all-approach is not applicable in the context of cancer care.

